# Extremophiles as a Model of a Natural Ecosystem: Transcriptional Coordination of Genes Reveals Distinct Selective Responses of Plants Under Climate Change Scenarios

**DOI:** 10.3389/fpls.2018.01376

**Published:** 2018-09-19

**Authors:** Stephanie K. Bajay, Mariana V. Cruz, Carla C. da Silva, Natália F. Murad, Marcelo M. Brandão, Anete P. de Souza

**Affiliations:** ^1^Center of Molecular Biology and Genetic Engineering, University of Campinas, Campinas, Brazil; ^2^Department of Plant Biology, Institute of Biology, University of Campinas, Campinas, Brazil

**Keywords:** adaptation, climate change, differential expression, gene co-expression network, mangrove, *Rhizophora mangle*, transcriptome

## Abstract

The goal of this research was to generate networks of co-expressed genes to explore the genomic responses of *Rhizophora mangle L.* populations to contrasting environments and to use gene network analysis to investigate their capacity for adaptation in the face of historical and future perturbations and climatic changes. RNA sequencing data were generated for *R. mangle* samples collected under field conditions from contrasting climate zones in the equatorial and subtropical regions of Brazil. A gene co-expression network was constructed using Pearson’s correlation coefficient, showing correlations among 78,364 transcriptionally coordinated genes. Each region exhibited two distinct network profiles; genes correlated with the oxidative stress response showed higher relative expression levels in subtropical samples than in equatorial samples, whereas genes correlated with the hyperosmotic salinity response, heat response and UV response had higher expression levels in the equatorial samples than in the subtropical samples. In total, 992 clusters had enriched ontology terms, which suggests that *R. mangle* is under higher stress in the equatorial region than in the subtropical region. Increased heat may thus pose a substantial risk to species diversity at the center of its distribution range in the Americas. This study, which was performed using trees in natural field conditions, allowed us to associate the specific responses of genes previously described in controlled environments with their responses to the local habitat where the species occurs. The study reveals the effects of contrasting environments on gene expression in *R. mangle*, shedding light on the different abiotic variables that may contribute to the genetic divergence previously described for the species through the use of simple sequence repeats (SSRs). These effects may result from two fundamental processes in evolution, namely, phenotypic plasticity and natural selection.

## Introduction

Brazilian mangroves represent the third largest area of mangroves in the world, covering 9,600 km^2^ (Giri et al., 2011). Given the wide extent and physiogeographic variation of the Brazilian coast, there is considerable regional diversity within the mangrove ecosystems. The tree composition (Angiosperms) of mangroves in Brazil is restricted to only six species belonging to the following three genera: *Rhizophora*, *Avicennia*, and *Laguncularia*. These plant species are considered extremophiles since they complete their life cycle in the presence of conditions that are extreme for most plants, such as muddy substrates with a low concentration of oxygen and periodic flooding by the tides, which causes large variations in salinity (Dassanayake et al., 2009).

*Rhizophora mangle* L. (Rhizophoraceae), popularly known as the red mangrove, is the most commonly found mangrove species in the Western Hemisphere in terms of density and distribution (Pil et al., 2011; Sandoval-Castro et al., 2012). The genetic variability of neutral molecular markers (simple sequence repeats; SSRs) in *R. mangle* reveals the presence of two large populations in Brazil; one population is found along the northern or equatorial coast, and another extends from the north-eastern extremity of South America to the state of Santa Catarina (SC) along the subtropical Brazilian coast (Pil et al., 2011; Francisco et al., 2018). There is greater genetic diversity between populations than within each population, which suggests that *R. mangle* along the Brazilian coast is not composed of a single panmictic population. The distinguishable allelic composition found in these two populations of *R. mangle* may result both from adaptation to local environmental characteristics and from stochastic factors such as genetic drift. Interestingly, the same pattern of spatial genetic structure is observed in neutral molecular markers in two other mangrove species, *Avicennia schaueriana* Stapf & Leechman ex Moldenke and *Avicennia germinans* L., both of which are dispersed by ocean currents (Mori et al., 2015) as well as in one mangrove-associated species, *Hibiscus pernambucensis* Arruda ex Bartol. (Takayama et al., 2008).

Recent genomic data have revealed a duplication of the complete genome of a *Rhizophora* species that occurred approximately 70 million years ago. These studies in comparative genomics also support the extremely low diversity of populations of *Rhizophora* and other mangrove species, revealing an evolutionary future threatened by the extreme conditions of intertidal zones. The investigation of genomic data is essential to the elucidation of the history and evolutionary mechanisms of adaptation that occur in parallel in each mangrove population (Xu et al., 2016). Despite the extensive presence of mangroves on tropical coasts, the lack of genetic diversity coupled with changes in sea levels make this ecosystem one of those most influenced by climate change in the current century (Loarie et al., 2009). The accelerated rates of climate change that have been observed will influence the distribution and development of mangroves and may lead to population reductions, accompanied by a reduction in the gene pool of the species, or in some locations, to range expansions (Alongi et al., 2000; Cohen et al., 2008, 2009; Krauss et al., 2008; Lara and Cohen, 2009).

The effects of these predicted scenarios on mangrove ecosystems must be carefully interpreted considering local environmental disturbances and the pressures on and characteristics of local populations. Overall increases in sea level and atmospheric temperature and changes in the frequency and intensity of rainfall events are predicted for both the equatorial and subtropical regions of the Brazilian coast (IPCC, 2014). In the face of changes in the frequency and intensity of rainfall as well as changes in temperature and sea levels, the salinity equilibrium in estuaries will also be altered (Short and Neckles, 1999; Alongi, 2008; Dolbeth et al., 2011). The Amazon River Basin is considered a hotspot for exposure to natural hazards (de Almeida et al., 2016) since significant changes in hydrology caused by rising temperatures (evaporation, stream flow and estuarine mixing of the sea and the river) will lead to more complex effects on ecosystem dynamics (Gillanders and Kingsford, 2002; Day et al., 2008; Milliman et al., 2008). Changes in the tidal regime, salinity and hydrology lead to changes in the structural development and distribution of estuarine species (Schaeffer-Novelli et al., 2002; Cunha-Lignon et al., 2011).

The potential for mangroves to adapt and survive depends on the physiological features of each species and their resilience and ability to withstand changes at the local and regional levels (Schaeffer-Novelli et al., 2016). Although climate change can be subtle and difficult to identify, it is expected to greatly affect coastal species at rates that may be faster than those that allow population adaptation. The resilience of mangrove populations is tied to multiple factors, including the genomic content of each species. This study was motivated by two studies of the genetic structure of Brazilian mangroves associated with fluctuating environmental conditions (Pil et al., 2011; Francisco et al., 2018). Investigating the non-neutral regions of the genome in the equatorial and subtropical subpopulations of the mangroves of South America may reveal important features of regional abiotic stress and, thus, help to predict their future in the face of climate change.

Our understanding of the complexity and molecular basis of mangrove tree adaptations has been enhanced by the generation of large amounts of genomic data, transcriptome data, annotated sequences, genetic components, natural selection data, and comparative genomic data (Dassanayake et al., 2009; Yang et al., 2015a,b; Li et al., 2016; Guo et al., 2017).

Our challenge here was to examine the gene transcripts from a mangrove tree species and to associate those transcripts with the available evidence of global climate change. Our study advances our understanding of the connection between environmental change and the genes that have the potential to respond to environmental pressures.

Research on individual genes cannot provide enough information to unravel the molecular mechanisms of stress resistance, which led us to conduct a natural laboratory experiment investigating the multigenic responses of mangrove plants immersed in their natural habitat. Through the presentation of a genetic correlation network obtained by examining the *de novo* transcriptome of *R. mangle L*., we described the patterns of gene expression in subpopulations located in the equatorial (Pará State) and subtropical (Santa Catarina State) regions of Brazil. The two populations of mangrove trees showed divergent networks of co-expressed genes, with transcripts clustered into two main sets. Inferences regarding the threat of climate change to each local population are discussed here.

## Materials and Methods

### Plant Materials

**FIGURE 1:**
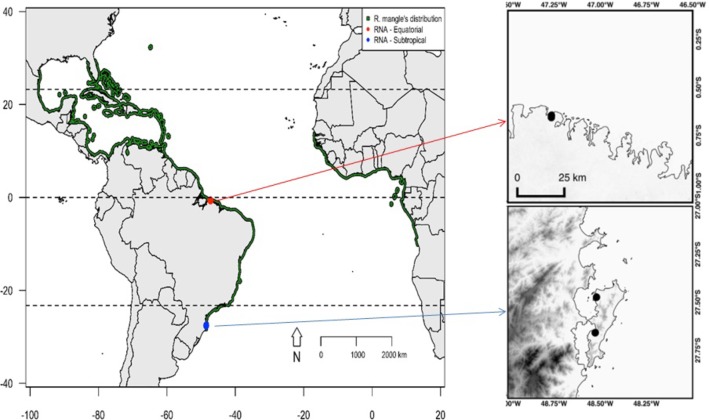
The distribution of *Rhizophora mangle* is indicated in green. Points represent the two plant material sampling sites. The red point represents the sampling site for the three plants in the equatorial region, and the blue point represents the sampling site for the three plants at the far southern end of the distribution of the species.

To identify the major environmental variables involved in the differential regulation of gene expression in *R. mangle* trees at contrasting latitudes, three adult plants (at reproductive age) were sampled from each of two natural populations under field conditions in the equatorial (municipality of Salinópolis, Pará State) and subtropical (municipality of Florianópolis, Santa Catarina State) regions of the Brazilian coast (**Figure 1**).

The equatorial sampling site is located in the Eastern American geographic region, where abiotic conditions are optimal for the occurrence of mangroves. During the collection of plant material at this sampling site, the forests were observed to be in advanced stages of development, with densely distributed trees, most of which were over 10 m tall. In contrast, the subtropical sampling site is at the southernmost edge of the distribution of the species in the Eastern American region; individuals in this population have a sparse distribution and rarely reach 10 m tall.

**FIGURE 2:**
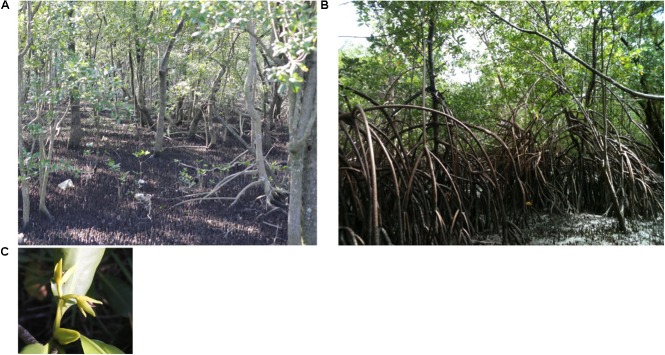
Phenotypic divergence between *Rhizophora mangle* L. at both sampling sites and species-specific floral traits. **(A)** A mangrove forest in the subtropical sampling site, where the distribution of individuals is sparse, and the prop roots of *R. mangle* do not reach 1 m in height. **(B)** An *R. mangle*-dominated forest along the equatorial coast of South America, where the prop roots reach up to 5 m in height. **(C)** A floral branch of *R. mangle*, showing the axillary inflorescence grouped into two branches, which is a characteristic used for species identification.

These sampling sites differ remarkably in the annual variation of key climate variables such as temperature, rainfall, relative humidity and insolation (**Figure 2**). Seasons are marked by dry and humid periods in the equatorial region, whereas cold and warm periods mark the seasonality in the subtropical region. The collection of plant material was conducted from July 26th to August 24th of 2014, at the beginning of the dry season at the equatorial site and at the end of winter at the subtropical site. To obtain a wide variety of *de novo* assembled transcripts, we sampled five distinct plant organs from each of the six collected plants, namely, the apical meristems of the branches, leaves, mature flowers, stems, and fine roots. As natural laboratories, these sampling sites provided ideal environments to examine the differences in gene expression that occur as a response in plants subjected to distinct historical stress conditions. Additionally, the equatorial site is located at the approximate distribution range center of *R. mangle* along the Atlantic coast of the American continent, and the subtropical site is located at the southernmost edge of its distribution range along the Atlantic coast of the American continent (**Figure 1**). Therefore, this experimental design allowed us to assess the environmental drivers of stress at the range center vs. the range edge.

Species-specific floral features were used to distinguish *R. mangle* L. from other sympatric congeneric species occurring on the equatorial coast. *R. mangle* has only 2 flowers on its floral branches, while *Rhizophora racemosa* G. Mey. and *Rhizophora harrisonii* Leechman have more flowers per branch (**Figure 2**).

Three individuals were sampled per sampling site (equatorial and subtropical) during low tide from areas with diverse physiognomies to characterize the general patterns of gene expression at each location. The trees were located in slightly flooded to moderately flooded areas and sandy to muddy soils. The position of each tree was recorded by a GPS receiver (Garmin: GPSMAP 76CS, Garmin International, Inc., Olathe, KS, United States). The sample coordinates, environmental data at the moment of sampling and characteristics of each sampled individual are available in **Supplementary Table 1** (RNA isolation and transcriptome sequencing).

Roots, stems, apical meristems, flowers and leaves were sampled under field conditions and immediately stored in individual Falcon tubes filled with an RNA stabilizing solution (RNAlater, Applied Biosystems/Ambion, Austin, TX, United States) and later processed in a laboratory. Total RNA was isolated from each sample using the Plant RNA Isolation Mini Kit (Agilent, Santa Clara, CA, United States). A NanoVue^TM^ device (Spectrophotometers Plus, GE Healthcare) was used to determine the RNA purity and concentration. To analyze the integrity of the samples, RNA was subjected to electrophoresis on a 1% agarose gel. Visualization of clear rRNA bands without traces (indicating degradation or contamination by genomic DNA) confirmed that the RNA quality was sufficiently high for the RNA to be used in cDNA sequencing.

To purify messenger RNA and produce cDNA, two 36-cycle paired-end TruSeq kits were used to prepare the libraries, followed by sequencing via 72 cycles on the Genome Analyzer IIx Illumina platform, according to the manufacturer’s instructions.

### *De novo* Sequence Assembly

Raw paired-end 72-bp reads were obtained from image data and transformed from base calling into raw sequence data. High-quality reads were filtered from the raw data through the removal of low-quality reads and adapter sequences using the *NGS QC Toolkit* (Patel and Jain, 2012). The clean reads were used in the *de novo* assembly of transcripts using Trinity v2.0.2 (Grabherr et al., 2011; Haas et al., 2013) and MIRA 4.0.1 (Chevreux et al., 1999). The minimum contiguous sequence (contig) size was set to 200 with at least 10x read coverage. A consensus transcriptome assembly was constructed based on the overlapping transcripts between the Mira and Trinity assemblies, using 100% BLASTN identity and 95% sequence overlap similarity as the thresholds (Camacho et al., 2009). In **Supplementary Data Sheet 1**, we include the manifest file used to run the MIRA software and the Trinity assembler command line to display command lines with default values and to specify any additional parameters used.

### Redundancy Filtering and the Identification of Putative Coding Sequences

After assembling the reads into longer contigs, we filtered the consensus assembly to reduce artefactual redundancy. Additionally, we identified the putative coding sequences (CDS) from the transcripts using the TransDecoder program (Haas et al., 2013). Similar transcripts were clustered with a customized Perl script, using a similarity threshold of 95% and requiring 90% of the length of the redundant sequence to align with the longest sequence. We retained only the transcript containing the longest putative CDS sequence present in each cluster.

### Annotation of Contigs

Functional annotation was performed using a customized set of Perl scripts and local databases built from publicly available data from the following databases: National Center for Biotechnology Information (NCBI) non-redundant protein and nucleotide databases (NR), Swiss–Prot^1^, and Gene Ontology (GO)^2^, with an *e*-value cut-off of 10e^-5^ and an HSP similarity threshold of 80%. The patterns of RNA families were determined using hmmscan (Eddy, 2011) with the RFAM database version 12 (Nawrocki et al., 2015). Protein families were obtained from the Pfam database (Finn et al., 2010) using translated peptides from putative coding regions determined by the TransDecoder program^3^.

### Detection of SSR Molecular Markers and Single Nucleotide Polymorphisms (SNPs)

Contigs larger than 1 kb were subjected to an SSR search with MISA software^4^. The detection of single nucleotide polymorphism (SNP) variants was performed with CLC Genomics Workbench 6.5.8 software, based on the Neighborhood Quality Standard (NQS).

### Differential Expression of Contigs

Bowtie 2 (Langmead et al., 2009) was used to map the high-quality paired-end reads from each sequence library to the transcriptome, and we recorded only the best alignment for each read. The ratio of reads mapped to the assembly is a useful criterion for evaluating the quality of the *de novo* assembled sequences. The number of reads mapped to each assembled contig was used for RNA-Seq expression analysis (Mortazavi et al., 2008).

The relative amount of each transcript in each sequence library was estimated using the RNA-Seq by Expectation Maximization (RSEM) program (Li and Dewey, 2011) using an expectation maximization (EM) algorithm that estimates maximum likelihood expression levels.

Differential expression analysis was conducted to compare the tissue-specific transcript expression profiles from the equatorial and subtropical samples. FPKM values (from RSEM) were used separately for each of the six sampled trees and their different plant organs (roots, stems, leaves, meristems, and flowers). This analysis was conducted in three distinct steps. The first step was to identify the uniquely expressed transcripts (UETs) at each sampling site (**Table 1**). The UETs were the transcripts expressed in all individuals and tissues from a given population that were absent from all samples from the other population. The second step was to select the equally expressed transcripts (EETs) among all three individuals sampled from each sampling site, to reduce the effects of local environmental variation on the gene expression in trees at the same sampling site. Only EETs present in all pairwise comparisons were used in the following step of the analysis to reduce the differences found among the individuals (genotypes) (**Supplementary Table 3**). The third step was to identify differentially expressed transcripts (DETs) between the overlapping EETs from the contrasting populations.

**Table 1:** Assembly parameters.

	Trinity	Mira	Mira + Trinity
Number of contigs	193,762	72,059	115,615
Longest contig	22,436	33,550	33,550
Shortest contig	224	149	149
Number of contigs > 1K nt	46,822 (34.0%)	16,300 (22.6%)	47,113 (40.7%)
Mean contig size	1,002	699	1,099
N50	1,680	1,350	1,740

**FIGURE 3:**
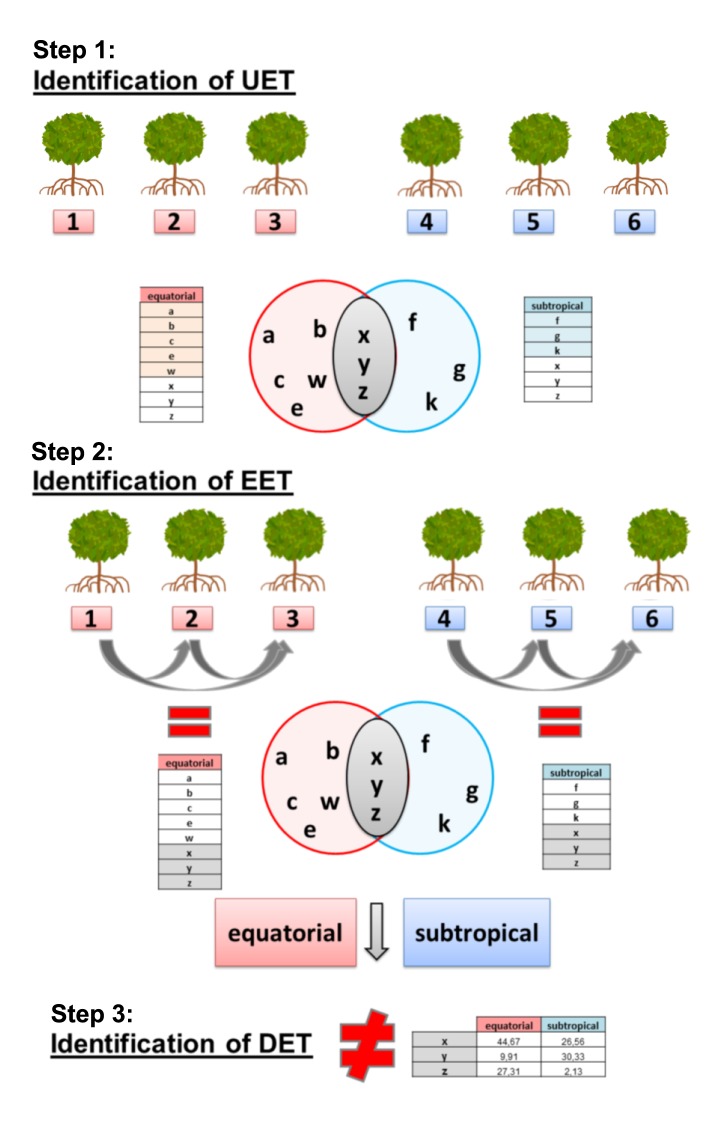
Differential expression analysis pipeline used in this work. (Step 1) Identification of uniquely expressed transcripts (UETs) in each population and subsequent selection of transcripts expressed in individuals from both populations to be used in step 2. (Step 2) Identification of equally expressed transcripts (EETs) (5% of false discovery rate) between each pair of individuals from each population and subsequent selection of transcripts expressed in individuals from both populations to be used in step 3. (Step 3) Identification of differentially expressed transcripts (DETs) (5% of false discovery rate) between the two populations.

This step was conducted using the posterior probability of equal expression (PPEE) value calculated with EBSeq (Anders and Huber, 2010), using an FDR < 0.05 and a fold-change > 1.5 as cut-off values. Thus, only transcripts that were expressed in all individuals from both populations were used in the differential expression analysis. The three individuals sampled from each location were treated as biological replicates after the selection of EETs (**Figure 3**).

### Validating Results: Quantitative RT-PCR Analysis

To validate the RNA-Seq experiment results, we used real-time reverse transcription qPCR (qRT-PCR) on ten reference genes and a total of fifteen DETs, with seven DETs from the leaves and eight DETs from the roots. The selected loci were homologous to the proteins described in **Supplementary Table 5** (>90% amino acid identity). Gene-specific primers were designed using Primer3Plus (Rozen and Skaletsky, 2000), with a target amplicon size of 70–150 bp and an optimal primer length of 20 bp. We performed qRT-PCR on the same RNA samples that were used for the RNA-Seq experiments. qRT-PCR amplification efficiency tests of all the primer pairs were performed using the reverse-transcribed mRNA (cDNA).

qRT-PCR was performed with a CFX384 Real-Time PCR Detection System with iTaq Universal SYBR^®^ Green Supermix (Bio-Rad Laboratories Inc., 850 Lincoln Centre Drive, CA, United States) following the manufacturer’s instructions, with a final primer concentration of 0.3 μM.

### Gene Co-expression Network

A single co-expression network was generated based on the EM values of all the transcripts from all the samples sequenced in the RNA-Seq experiment. Transcripts showing > 50% null values in the samples were excluded to reduce noise and eliminate residuals in the analysis. Following noise removal, the Pearson’s correlation values between each pair of transcripts for each plant organ (roots, stems, leaves, meristems, and flowers) were used to construct a co-expression network. Transcripts in the network were clustered using the Heuristic Cluster Chiseling Algorithm (HCCA). The highest reciprocal classification (HRR) method proposed by Mutwil et al. (2009) was employed to select only edges representing the strongest correlations (VALUE ≤ 30), where an HRR of less than or equal to 3 was used to empirically filter the edges.

The transcript co-expression network was independently analyzed for each plant organ, based on the annotation of transcripts and their tissue-specific expression levels across samples. DETs between equatorial and subtropical populations, UETs from each population and their first neighbors were highlighted in different colors in the network. Cytoscape 3.4.0 software (National Institute of Medical Sciences)^5^ was used to visualize the tissue-specific transcript expression classes (DET, UET and neighbors). For data handling and interpretation of the results, clusters containing transcripts associated with stress responses were selected. The enrichment of GO terms in the network was analyzed using the R package *GOseq* (Young et al., 2010).

Analyses of the GO terms most highly represented in the tissue-specific networks were conducted for the following three sets of transcripts: DETs, UETs and EETs. Additionally, enrichment analysis was performed for each of the five clusters comprising most of the transcripts in each network (*p*-values of a pathway less than 0.05) (**Supplementary Figures 5**, **6**).

## Results

### *De novo* Assembly of the *R. mangle* Reference Transcriptome

The combined assembly containing the contigs that overlapped between the Trinity and Mira *de novo* assemblies had better metrics than the isolated assemblies from each software (**Table 1**). The mapping of the total set of reads to the selected assembly was very high (96.87%). A similar proportion of reads obtained from the samples from each population mapped back to the final assembly, with 97.03 and 96.68% of the reads from the subtropical and equatorial population samples mapped, respectively.

The completeness of the combined assembly was assessed by comparing the transcripts to the Benchmarking Universal Single-Copy Orthologs (BUSCO) plant database (Simão et al., 2015). BUSCO performs a quantitative analysis of how complete an assembly is based on evolutionary expectations of the content of universal single-copy orthologous genes (present in approximately 90% of plants). The *R. mangle de novo* assembly characterized herein recovered the majority orthologous sequences from the BUSCO plant dataset (97.1%), of which 94% were complete and 2.4% were fragmented sequences.

**FIGURE 4:**
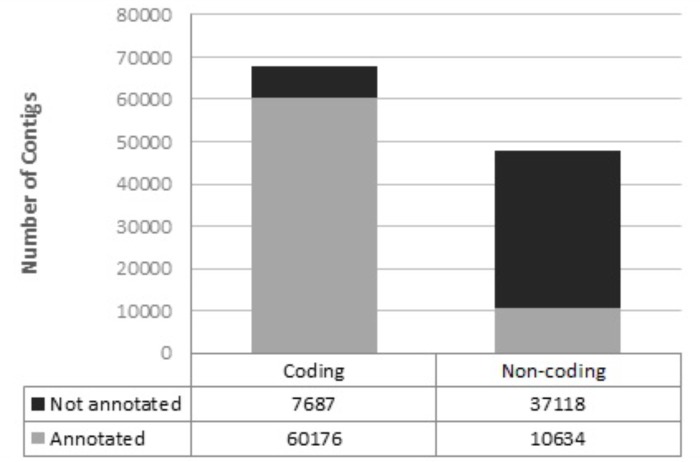
Annotation of assembled transcripts using a customized perl script and several databases as a reference.

In total, 115,615 transcripts were assembled, 69,452 of which contained a reading frame for protein synthesis (open reading frame, ORF). Most of the putative coding transcripts were annotated, while most of the putative non-coding transcripts were not annotated (**Figure 4**).

### Functional Annotation

**FIGURE 5:**
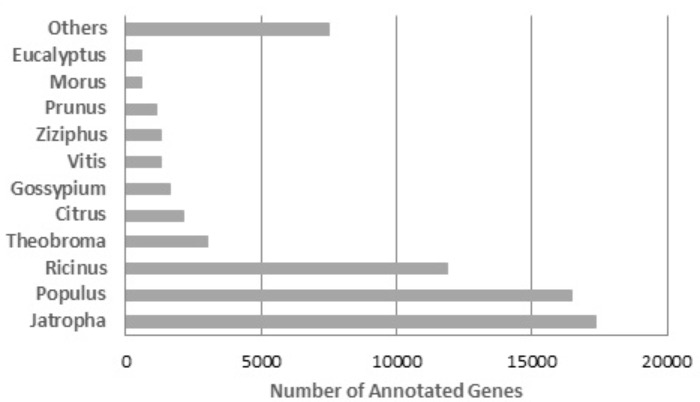
BLASTx top-hit species distribution of gene annotations using plant RefSeq as a reference.

The assembly was blast-aligned against the NCBI Plant RefSeq Database, and 64,867 (56.1%) contigs were annotated. When the annotation redundancies were removed from each contig, 54,058 contigs (46.75%) with unique annotations were obtained. The species that presented the highest number of sequences homologous to the *R. mangle* contigs was *Jatropha curcas* with 17,376 contigs, followed by *Ricinus communis* with 11,863 contigs and *Populus trichocarpa* with 8,534 contigs (**Figure 5**). The high degree of similarity between the transcripts from *R. mangle* and these three species is expected since all four species belong to the order *Malpighiales*. Based on the phylogeny of these species, they are taxonomically close to *R. mangle*. This result is evidence that the transcripts of this assembly were very specific, meaning that the filtering of non-specific transcripts was efficient.

The annotation using the complete database (described in the Section “Materials and Methods”) returned 70,810 transcripts that were homologous to previously described protein sequences. Only 44,805 of the transcripts had no similarity to any sequence present in the database. In the GO analysis, we found 223,743 orthologous genes associated with 44,481 transcripts from the assembly. Of the GO terms identified, 53,767 were associated with the hierarchical category of cellular components, 79,746 were associated with molecular functions, and 90,230 were associated with biological processes (**Supplementary Figure 1**).

Compared with the transcriptome previously developed for *R. mangle* that was sequenced using a 454/Roche GSFLX platform (Roche Applied Science, Indianapolis, IN, United States) (Dassanayake et al., 2009), the assembly in this study contained 10,048 new contigs. Out of the 44,481 annotated contigs from our transcriptome, 34,433 contigs were homologous to the previously reported transcriptome.

### Detection of SSRs and SNPs

It was possible to identify 14,283 SNPs in the transcribed regions. More SNPs were found in the equatorial samples (14,283) than in the subtropical samples (8,875), when reads from the equatorial and subtropical regions were mapped separately. On average, 10,000 SNPs were common between the regions (10,416 between equatorial samples and the total assembly and 9,690 between subtropical samples and the total assembly). Unique SNPs were also identified; 5933 were found in the equatorial samples, and 2,020 were found in the subtropical samples.

The larger number of SNP markers identified in the transcriptome of the equatorial samples agrees with the greater genetic diversity found along the equatorial coast in previous works using neutral SSRs in *R. mangle* (Francisco et al., 2018). This high genomic diversity in the equatorial region may be associated with the colonization history of the species or with the environmental divergence between the equatorial region and the subtropical region. Populations in the range center (equatorial region) could face more relaxed selective pressures than populations along the southern margin of the range, where environmental conditions could be suboptimal for the occurrence of the species. In this way, the identification of polymorphic loci within the transcribed regions of the genome is a valuable tool for evolutionary studies.

The number of SSRs in the subtropical samples (34,289) was similar to that in the equatorial samples (34,301), which is expected because both populations belong to a single species.

### Gene Co-expression Network

#### Differential Expression

Principal component analysis (PCA) was performed using normalized expression data obtained from RSEM. In a preliminary PCA conducted for all 30 samples (five distinct tissues from six distinct individuals), samples from the same tissues clustered together. We then conducted five tissue-specific PCAs, in which samples from distinct sampling sites did not cluster together (**Supplementary Figure 4**). This finding led us to adopt additional steps in our strategy for the identification of DETs, as we described in the Section “Materials and Methods.”

The number of UETs per region varied between tissues (**Supplementary Table 2**). We analyzed only UETs directly connected to DETs in the network (**Table 2**). Thus, we focused only on the UETs associated with the differential expression found between the populations. For all tissues except the roots, we found more genes with higher relative expression levels in the equatorial samples than in the subtropical samples.

**Table 2:** Number of DETs in the network and the first-level neighbors that are UETs.

Tissue	Over-expressed in equatorial samples	Over-expressed in subtropical samples	UETs in subtropical samples	UETs in equatorial samples
Flower	202	94	164	174
Leaf	192	26	178	184
Meristem	181	27	52	190
Root	83	123	40	332
Stem	185	75	196	340

It is expected that *R. mangle* individuals at the equatorial region are exposed to a more relaxed environment in terms of selective pressures in comparison to individuals living on the periphery of the species distribution, such as the subtropical zone from which plant material was sampled in this study. Stress increases as the distance from the equator increases, and one or more factors may limit the occurrence of the species.

#### Co-expression Network Structure

To visualize the relationships among genes in a co-expression network, pairwise Pearson’s correlations were calculated. To simplify the analysis of differential expression between samples from distinct populations, only the first-level neighbor of each DET was chosen. The co-expression analysis was performed individually for each sampled tissue to identify the complete network of transcripts expressed in that tissue. A test was performed on the stem network to increase the correlation level to the second neighbor of each DET. The result was that a network of 3,608 genes grew to 21,512 genes. Although the correlations among the transcripts in the network structure remained the same, increasing the number of neighbors in the differential expression analyses precluded the functional interpretation of the enrichment results for GO terms.

**FIGURE 6:**
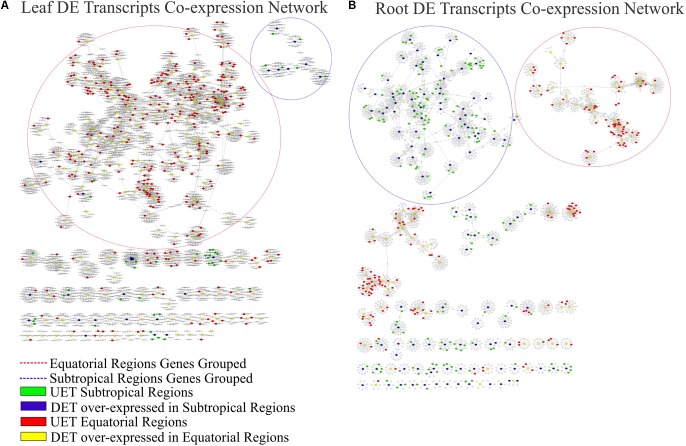
Correlation networks of transcript expression in *Rhizophora mangle*. Circular nodes represent transcripts and edges represent the correlation between pairs of transcripts. The figures show differentially expressed transcripts (DETs) between populations and their first neighbors. A blue circle highlights one of the largest clusters of these networks, with a predominance of UETs and over-expressed transcripts detected in subtropical samples. A red circle highlights one of the largest clusters of these networks, with a predominance of UETs and over-expressed transcripts detected in equatorial samples. **(A)** Co-expression network of leaf transcripts. **(B)** Co-expression network of root transcripts.

**FIGURE 7:**
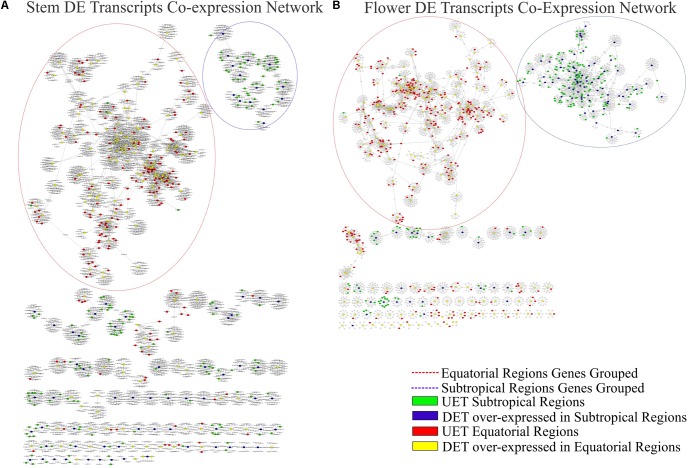
Correlation networks of transcript expression in *Rhizophora mangle*. Circular nodes represent transcripts and edges represent the correlation between pairs of transcripts. The figures show differentially expressed transcripts (DETs) between populations and their first neighbors. A blue circle highlights one of the largest clusters of these networks, with a predominance of UETs and over-expressed transcripts detected in subtropical samples. A red circle highlights one of the largest clusters of these networks, with a predominance of UETs and over-expressed transcripts detected in equatorial samples. **(A)** Co-expression network of stem transcripts. **(B)** Co-expression network of flower transcripts.

**FIGURE 8:**
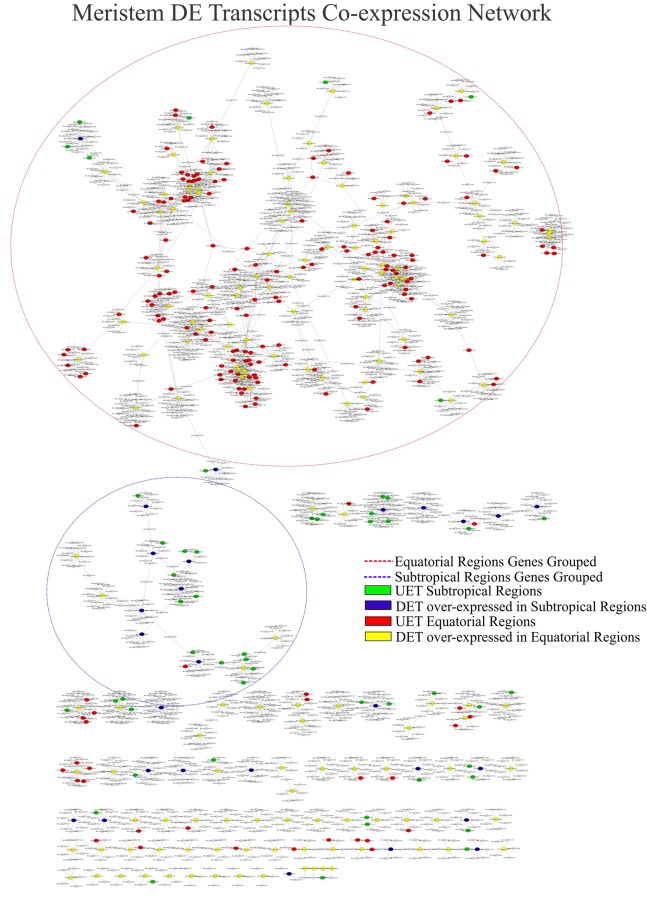
Correlation networks of meristem transcript expression in *Rhizophora mangle*. Circular nodes represent transcripts and edges represent the correlation between pairs of transcripts. The figures show differentially expressed transcripts (DETs) between populations and their first neighbors. A blue circle highlights one of the largest clusters of these networks, with a predominance of UETs and over-expressed transcripts detected in subtropical samples. A red circle highlights one of the largest clusters of these networks, with a predominance of UETs and over-expressed transcripts detected in equatorial samples.

The complete co-expression network had 78,848 transcripts correlated across 550,916 arcs, with a total of 992 clusters. When the tissue-specific analysis was performed, the partial networks of all five tissues exhibited two major clusters marked by the presence of the following: (1) DETs over-expressed in the subtropical samples and their correlated transcripts and (2) DETs over-expressed in the equatorial samples and their correlated transcripts (**Figures 6–8**). UETs in each sampling population were frequently observed to have a first-level correlation with a DET with a higher expression level in the same sampling population.

This method of visualizing the date highlights the differences in expression and the importance of genes that had no difference in expression but might play a role in the regulation of DET genes.

#### Enrichment Analysis of GO Terms

The enrichment analysis showed 84 GO terms in both major clusters of the gene-correlation network containing DETs over-expressed in the equatorial samples and DETs over-expressed in the subtropical samples. However, the set of transcripts over-expressed in the equatorial samples was larger than the set of transcripts over-expressed in the subtropical samples for the leaves, meristems, stems and flowers. Only roots had more DETs that were over-expressed in the subtropical samples than in the equatorial samples.

The five sub-clusters that included the highest number of transcripts were analyzed for each tissue-specific co-expression network (**Table 3**). Some of these most representative clusters were frequently found in more than one tissue-specific network. This result means that these clusters contained DETs and their correlated transcripts that are substantially relevant to population-level differentiation.

**Table 3:** The five most representative clusters of each network and selected GO term enrichment.

Cluster ID	Tissue	Number of genes in cluster	Number of genes in module (network)	Enriched biological processes (GO terms)
17	Stem	528	72	Response to absence of light; response to ethylene; response to auxin
226	Stem	486	69	Oxidation-reduction process; cellular response to stress; sodium ion transport; cell redox homeostasis; oxidation-reduction process
248	Stem	93	69	Phloem or xylem histogenesis; transmembrane transport
11	Stem	244	67	Transmembrane transport; proteolysis; phosphorylation; cell wall biogenesis; transport; response to salt stress; response to heat; response to oxidative stress; response to stress
179	Stem	109	58	Ion transport; sodium ion transmembrane transport; translation
98	Leaf	362	92	Response to wounding; response to freezing; response to gamma radiation; response to high light intensity
179	Leaf	109	68	Regulation of pH; response to salt stress
147	Leaf	97	48	Golgi to plasma membrane transport; lipid metabolic process; response to chitin
392	Leaf	143	48	Response to UV-B
17	Leaf	528	45	Response to water deprivation; hyperosmotic salinity response
70	Flower	63	82	UDP-glucose transport; regulation of transcription, DNA-templated
292	Flower	125	78	Nitrogen compound metabolic process; floral organ formation; regulation of flower development; vegetative to reproductive phase transition of meristem
42	Flower	191	70	Pollen tube growth; pollen development
57	Flower	288	68	Biosynthetic process; response to oxidative stress; oxidation-reduction process
248	Flower	93	64	Oxidation-reduction process
110	Meristem	144	53	Response to osmotic stress; response to cyclopentenone
392	Meristem	143	53	Anthocyanin accumulation in tissues in response to UV light
241	Meristem	94	49	Transport
179	Meristem	109	47	Gene silencing by miRNA; cellular amino acid metabolic process
248	Meristem	93	44	Oxidation-reduction process; production of miRNAs involved in gene silencing by miRNA
302	Root	160	83	Response to oxidative stress; oxidation-reduction process
60	Root	146	69	Transmembrane transport
32	Root	321	61	Regulation of nucleic acid-templated transcription; biosynthetic process
91	Root	903	57	Heat acclimation; MAPK cascade; response to salt stress; response to UV-B; response to far-red light; response to red light; response to high light intensity; response to blue light; response to light stimulus; response to heat; cellular response to light intensity
18	Root	143	55	Intracellular signal transduction

*The GO terms shown here were selected based on the climate differences between sampling sites*.

The classification of UETs in the subtropical or equatorial samples into ontology terms frequently revealed the same categories, such as “regulation of pH,” “response to salt stress,” “oxidation-reduction process”; “cellular response to stress” and “response to heat.” However, it is clear that the trees had different sets of transcripts that were responding to similar abiotic conditions found in each of the sampling sites. The transcript correlations were distinguishable between the subtropical and equatorial samples.

### Validation of the Differential Expression Results

To confirm the results of the RNA-Seq analysis, we determined and statistically analyzed the transcription levels of selected genes using RT-qPCR, including 15 variable genes and 10 invariable genes. The results were strongly consistent between the two methods, with 84% of the genes tested having similar expression profiles and similar categories (invariable and variable genes). Eleven out of 15 primer pairs had amplification efficiencies between 90 and 110% and were, therefore, the only primer pairs used for validation (**Supplementary Table 5**). The putative housekeeping genes selected as reference genes in the relative expression analyses are described in **Supplementary Table 4**.

Eight of the primer pairs yielded qPCR results that confirmed the differential expression identified by EBSeq (**Supplementary Figures 2**, **3**), and the difference was confirmed by Student’s *t*-test (*p*-value < 0.05). The remaining primers showed statistically significant differences between the levels of expression in the equatorial and subtropical regions [primers DBR (leaf), CesA (leaf) and GID1B (root)] (**Supplementary Figures 2**, **3**).

## Discussion

We performed a *de novo* assembly of the *R. mangle* transcriptome using high-throughput sequencing of 30 cDNA libraries obtained from RNA extracted from five plant organs (apical meristems, leaves, stems, roots, and flowers) that were sampled from six individuals. Even though this was not the first transcriptome assembled for *R. mangle* (Dassanayake et al., 2009), the final high-quality transcriptome presented here contains a wider representation of the species’ transcripts than did the first published transcriptome for the species, including over 10 thousand transcribed sequences reported for the first time and over 97% of the universal orthologous plant sequences present in the OrthoDB. Additionally, we were able to annotate nearly 90% of the putative protein coding transcripts in the assembly using distinct databases, such as the non-redundant database from NCBI, the plant RefSeq database from NCBI and the Pfam database, as references. Based on their annotation, transcripts were classified into functional categories from the GO Consortium. We detected SSRs and putative SNP sites present in the transcribed sequences, which may be useful resources for further diversity studies of *R. mangle*.

The larger number of SNP markers identified in the transcriptome of the equatorial samples than in that of the subtropical samples agrees with the greater genetic diversity found along the equatorial coast than in the subtropical region in a previously published study using neutral SSRs from *R. mangle* (Francisco et al., 2018). The high degree of genomic diversity in the equatorial region may be associated with the colonization history of the species or the divergence in climate between the equatorial region and the subtropical region. The equatorial mangrove population could face more relaxed selective pressures than the mangrove population at the southern edge of the distribution range, where environmental conditions may be suboptimal for the survival of the species. For this reason, the identification of polymorphic loci within transcribed regions of the genome is a valuable tool for evolutionary studies.

The number of SSRs in the subtropical samples (34,289) was similar to that in the equatorial samples (34,301), which is expected because both populations belong to a single species.

Differentially expressed transcript analysis showed that there was a higher abundance of transcripts associated with photosynthesis, chlorophyll and flavonoid biosynthesis, cellular respiration, photorespiration, and the cellular responses to starvation, high temperature, pectin and cellulose breakdown and disease in samples from the equatorial region than in those from the subtropical region. In contrast, compared with samples from the equatorial region, samples from the subtropical region was a higher abundance of transcripts associated with photosynthetic acclimation, the cellular response to high light conditions and starch biosynthesis. The clustering pattern can be explained by the distinct abiotic conditions to which the samples were exposed at the moment of sample collection (**Supplementary Table 1**), but historical adaptive variation in the regulation of gene expression may also play a role.

The higher number of over-expressed genes in the subtropical samples than in the equatorial samples may result from a negative regulation of gene expression in response to increased environmental stress. In the equatorial region leaf samples, for example, we observed over-expression of heat shock proteins (Hsp90.2) that can be instantly inactivated when the plant experiences thermal shock (Yamada et al., 2007). In addition, the high expression levels of ethylene response factors (ERFs) observed in equatorial samples are associated with the activation of all stress response signaling cascades (Müller and Munné-Bosch, 2015).

The lower level of the transcription of stress genes in the subtropical region than in the equatorial region may be corroborated by previous studies that report mangrove expansion toward higher latitudes at the southern boundary in Brazil in response to global climate change. The lower expression levels of putative stress response genes in samples from the subtropical region than in samples from the equatorial region may be the result of the ancient colonization of genotypes that support the conditions of a more temperate region.

**FIGURE 9:**
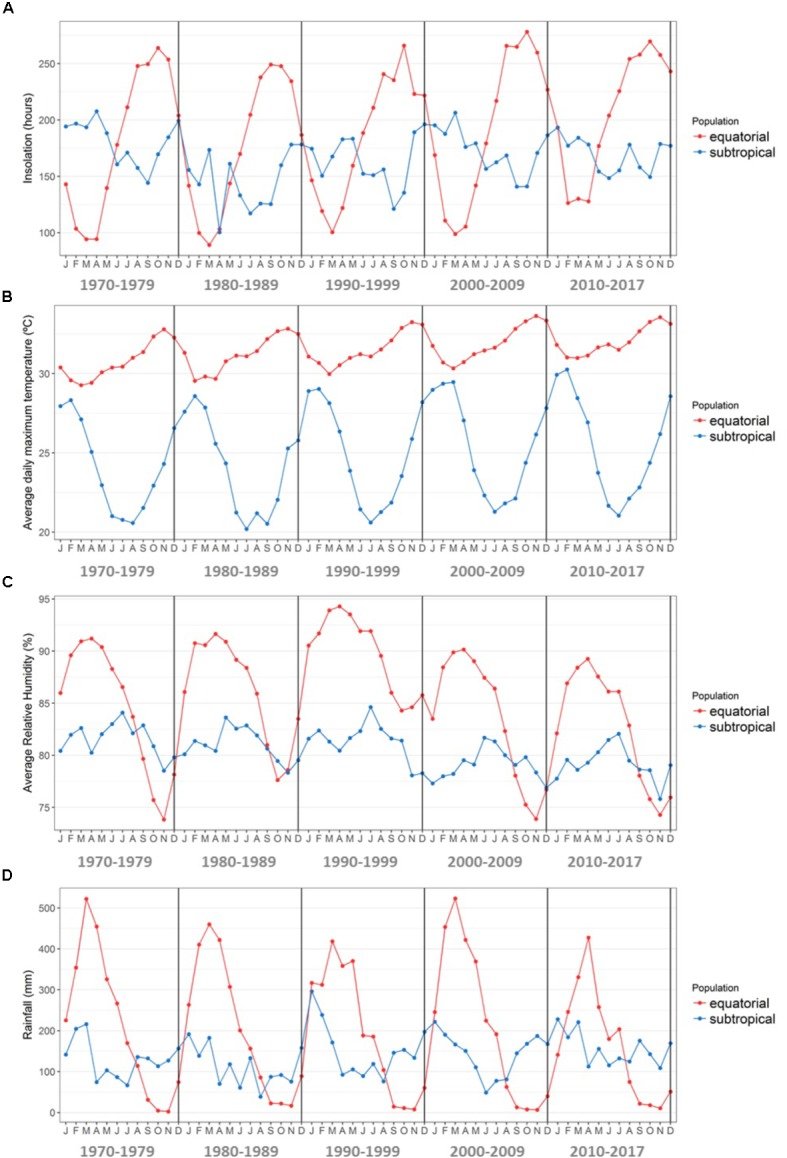
Climate characterization during the period 1970–2017 in the equatorial and subtropical sampling sites. Data were downloaded from the Tracuateua (1.06°S/46.90°W) and Florianópolis (27.58°S/48.56°W) automatic weather stations of the Brazilian National Institute of Meteorology (INMET) website. **(A)** Average monthly insolation in each decade (hours). **(B)** Average daily maximum temperature per month in each decade (°C). **(C)** Average monthly atmospheric relative humidity in each decade (%). **(D)** Average monthly rainfall in each decade (mm).

The interpretation of the data is based on the analysis of the transcriptional patterns found in gene co-expression networks and their relationships with the known climatic variables of the two sampling sites (**Figure 9**). The genetic functions and the processes in which transcripts are involved are revealed by the enriched network clusters in addition to the DETs. The co-expression network allowed a broader view of the transcriptional differences between samples from the equatorial and subtropical regions since, in addition to the DETs, the neighboring genes were also considered with regard to the enrichment of the representative gene categories. This analysis enabled a more comprehensive analysis of all the transcripts obtained in the *R. mangle* transcriptome that potentially participate in the regulation of the responses of the plant to contrasting environmental conditions.

Our results reveal the effects of contrasting environments on gene expression in *R. mangle*, shedding light on the different abiotic variables that may contribute to the genetic divergence previously described in the species by the use of SSR markers. These effects may be the result of two fundamental processes for adaptation, phenotypic plasticity and natural selection. These results are similar to those of previous genomic studies that demonstrated amino acid (AA) changes in the *Rhizophoras* genome, suggesting local adaptations of the functional genome in different populations of these trees. The frequent substitution of AAs suggested a rapid evolution of the proteins in mangroves. These proteins are associated with highly specialized mangrove traits for survival in intertidal habitats. The parallel evolution of the functional genomics of these mangrove species is driven by Darwinian selection (Xu et al., 2017).

We used the assembled *de novo* transcriptome to map reads and to estimate the abundance of each assembled transcript in each of the sequenced libraries. The estimations of abundance were confirmed through qRT-PCR with cDNA obtained from the same RNA samples that were used in sequencing as a template (**Supplementary Figures 2**, **3**). The consistency between the estimation of the abundance of transcripts from the equatorial and subtropical samples by qRT-PCR and the expression values of the sequenced transcripts confirmed the robustness of the analysis. Our transcriptome can be used to select candidate genes for further studies on the response to heat in *R. mangle* and its possible correlation with adaptive selection.

The transcriptionally stable genes we adopted in our RT-PCRs may also serve as reference genes for other studies. The correct selection of highly stable genes for each tissue studied is extremely important for the accurate analysis of the genes chosen to estimate differences in the abundances of the transcripts (Pombo et al., 2017).

Following confirmation of the RNA-Seq results by qRT-PCR, the transcripts abundance data were used as the input for both the construction of a co-expression network, based on the pairwise Pearson’s correlations of the transcripts, and for the comparison of transcript expression levels between the two natural populations of the species, one located at the range center and one at the southern edge of the range of the species. In the differential expression analysis, we compared trees sampled under field conditions from contrasting latitudes in the equatorial and subtropical regions of the Atlantic coast of South America (see the Materials and Methods). Focusing our analyses on the differences in abiotic factors at these two sites, which were possibly associated with the observed differential regulation of transcript expression levels in the sequenced samples, we found some interesting results that were corroborated by the structure of the co-expression network (see **Figures 6–8**). The correlation network of the transcripts with different expression levels between the two populations indicated that different groups of putative genes may be involved in the response to divergent stress factors in these two sites. Predicting the effects of climate change on natural populations is challenging, especially when other global issues are also considered, such as increasing sea pollution and the increasing human population (Rockström et al., 2009). To simplify this complex scenario, we considered only key environmental variables that control the global distribution of the mangrove species (Osland et al., 2017) (shown in **Figures 9B–D**) when analyzing the transcript expression data from the equatorial and subtropical *R. mangle* samples. The following variables were considered: temperature, rainfall, adaptation to low oxygen levels (flooding), limited salt levels and the associated water loss, increased survival of offspring, and mangrove conservation.

### Temperature Stress Response and Global Warming

By the end of the 21st century, an increase of 3–5°C in the annual mean air temperature is expected by the National Centers for Environmental Information (NCEI).

There is evidence in the literature of mangroves expanding poleward to their latitudinal limits (Saintilan et al., 2014), such as *Avicennia marina*, which expanded its distribution in southern Africa. On the eastern coast of the United States*, R. mangle, Laguncularia racemosa* and *Avicennia germinans* are also rapidly expanding their ranges to higher latitudes (Cavanaugh et al., 2015). Additionally, Osland et al. (2017) expects that mangroves at the southernmost limit of the eastern South American coast will expand southward, even though there is no evidence of recent expansion in the literature (Soares et al., 2012). The seasonal variation in air temperature increases from the equatorial to subtropical regions. Average temperatures during the day are generally above 30°C in the equatorial region of the Brazilian coast. Thus, continuous warming, especially in the tropical ecoregions could negatively impact mangroves due to thermal stress. Consistent with this argument, cluster 392 from the gene expression network (**Supplementary Figure 5**) showed that the transcript expression levels of genes involved in the responses to heat were higher in the equatorial samples than in the subtropical samples.

For instance, a transcript similar to “heat stress transcription factor B-2B” and a transcript containing a “heat shock protein 9/12 domain” were exclusively expressed in all tissues sampled from plants from the equatorial region. Additionally, we found transcripts that were uniquely expressed in three different tissues in the equatorial samples that showed similarity to “heat shock protein 82,” “heat shock protein 83-like,” and “heat shock 70-kDa protein cognate 4.”

Cluster 226 (**Table 3**) from the stem network, for example, contained transcripts that were correlated to UETs and DETs with higher expression levels in the equatorial samples than in the subtropical samples. These transcripts were associated with the GO term “response to heat” (GO: 0009408), demonstrating the correlation among putative genes responding to the same biological process. The over-expression of heat shock proteins in the equatorial samples could correlate with molecular chaperones involved in RPM1-mediated resistance. Components of the RPM1/RAR1/SGT1 complex, these super DETs may stabilize RPM1 and protect it from SGT1-mediated degradation. These super DET proteins associate with RAR1, which possesses ATPase activity and may function as a co-chaperone (Hubert et al., 2003, 2009). In the absence of heat shock, RAR1 negatively regulates heat-inducible genes by actively suppressing the heat acclimation function of heat shock transcription factor A1D (HSFA1D) (Yamada et al., 2007). All these chaperone mechanisms also involve the induction of the expression of heat shock transcription factor A2 (HSFA2) in response to oxidative stress (Nishizawa-Yokoi et al., 2010). The high expression level of heat shock proteins in the younger stems also correlates with the expression of heat-inducible genes and acclimation to excess heat, inducing the closure of stomata and modulating transcriptional and physiological responses to abscisic acid (ABA) (Clément et al., 2011). Heat shock proteins regulate RPP4-mediated temperature-dependent cell death and defense responses (Bao et al., 2014). These super DETs that are heat shock proteins may assist SGT1B in the formation of SCF E3 ubiquitin ligase complexes that target the immune receptors SNC1, RPS2 and RPS4 for degradation to regulate receptor levels and avoid autoimmunity (Huang et al., 2014).

The higher expression levels of putative disease responsive genes in the equatorial samples than in the subtropical samples may be associated with high temperature. Heat can enhance the incidence and severity of diseases, as well as the susceptibility of the host to disease. For example, high temperature can increase stem rust and leaf rust because high temperatures are optimal for pathogen growth (Dawson et al., 2015).

Cluster 302 showed several differential UETs and DETs with higher expression levels in the roots of the equatorial samples than in the roots of the subtropical samples, including putative genes associated with “protein refolding” (GO: 0042026) and “response to UV” (GO: 0009411).

The presence of this protein refolding and response to UV term in many clusters and in several tissues may be associated with its participation in complex signaling pathways and various regulatory processes in plant cells, including responses to environmental stress, such as temperature (Mishra et al., 2006).

“Proteolysis” (GO: 0006508) is a GO term that was present in several clusters (clusters 11 and 226 of stems, 110 of meristems, 392 of leaves and 57 of flowers), and it plays an important role in the trafficking of hydrolysed proteins, balancing the recovery from stress and excessive protein degradation that results in cell death (Diaz-Mendoza et al., 2016). This protein degradation may be caused by excess heat or a response to pathogens.

Cluster 98 was present in leaf genes in the equatorial samples and was associated with the GO terms “macromolecule depalmitoylation,” “response to high light intensity,” and “shikimate metabolism,” which are related to the response to high-intensity UV radiation (Tzin and Galili, 2010). Another GO term that was also present in this cluster, “pectin biosynthesis” (GO: 0045489), is also involved in the response to different stresses (Houston et al., 2016). The term “response to UV-B” also appeared in genes in cluster 329 in leaf samples from the equatorial site.

In cluster 98, in samples from the subtropical region, we also found the enrichment of genes related to the response to freezing, which is expected since the current geographic range of mangrove indicates that excessive cold in a saline environment can severely limit the provision of water to the leaves (Stuart et al., 2007). In addition to this adaptation to the cold, the subtropical plants showed an over-expression of genes related to heat response and light intensity, such as the upregulated DET “photosystem II reaction center W protein” in three of the five tissues (meristem, leaf, and flower) or the highly expressed UET of “cryptochrome DASH.” High temperature stress induces oxidative damage to proteins present in photosystem II (PSII) (Yamamoto, 2016), which may lead to changes in the levels of proteins involved in PSII.

This adjustment of the different selective pressures seems to be an advantage for subtropical mangrove plants. The absence of adaptive adjustments may be a problem for plants in the equatorial region, which, similar to other basic plant species, must direct most of their efforts to adapting to high temperatures. There may be declines in the yields of tropical plants and increases in the yields of temperate plants that seem to apply to the survival of the mangrove (Zhao et al., 2017; Scheelbeek et al., 2018).

Cluster 32 was associated with many genes that were DETs and UETs in the root samples from the subtropical population and with “non-photochemical quenching,” which is linked to the essential regulation of a high light stress photoprotection mechanism. This response in subtropical root samples may be associated with the continuous exposure of the roots to light when the level of the incoming tide is low (Lambrev et al., 2012). There were also genes linked to the GO term “maltose metabolism,” which is related to stress response to acute temperatures, such as cold (Kaplan and Guy, 2004). These characteristics are important for the current weather conditions in the subtropical region, which experiences cold seasons and is predicted to experience increased temperatures.

High temperatures adversely affect the development of plants in many ways (Sánchez et al., 2014). Current temperatures are already activating a gene pool related to the response to higher temperature stress in equatorial plants but not in subtropical plants. With continued warming under ongoing greenhouse gas emissions, these populations are expected to decrease and become more varied, as is also predicted for other plants in the face of future warming scenarios (Tigchelaar et al., 2018).

### Changes in the Rainfall Regime and Climate Change

Mangrove forests in the humid tropics have higher biomass and productivity than those in areas that are more arid or have less rainfall, such as subtropical Brazil (Alongi, 2015). The rainfall deficit and high salinity predicted for northern (equatorial) Brazil could lead to the loss of mangrove areas due to reduced primary productivity and seedling survival and changes in interspecific competition. These systems are already facing less rainfall and a decreased frequency of extreme precipitation events due to the inter-annual climate variability that is strongly driven by El Niño-Southern Oscillation (Grimm and Tedeschi, 2009).

In addition, precipitation patterns may also greatly affect the development of pathogenic fungal disease, as an excess or scarcity of relative air humidity is required by most fungal infections (Steffenson, 2003). It is possible to find pathogen response enriched GO terms in the genes of the *R. mangle* network, such as “defense response” (GO: 0006952) in cluster 11 of the stems and cluster 147 of the leaves. The upregulation of genes involved in the response to pathogens in equatorial samples is related to high temperatures that provide a favorable environment for the growth of stem and leaf fungi.

Equatorial samples were more efficient at vacuolar accumulation of Na+, with upregulated expression of H+-ATPase (ATPase H+) genes. Vacuolar accumulation of Na+ is an important tool for maintaining the homeostasis of the ions inside the cell in the mangroves (Parida and Jha, 2010). The cell walls in equatorial plants respond to the current high levels of rainfall; however, in the future, they will have to respond to the expected drought in the region. Water deficits and higher salinity can lead to increased evapotranspiration and decreased primary productivity. These metabolic responses can completely alter how *R. mangle* grows as well as its phenology (Alongi, 2015). In subtropical Brazil, where precipitation rates are expected to increase, the radicular system, which is relatively shallow, may be more vulnerable to wave action (Schaeffer-Novelli et al., 2002). There was a set of genes among the UETs that were responsive to auxin, an important plant hormone that provides a key signal for the formation of lateral roots in many plants. Auxin maintains the homeostasis and directional transport of ions and is essential for plant growth and development (Krishnamurthy et al., 2017). Among the auxin-responsive UETs found in the samples from the subtropical region were the following: “indole-3-acetic acid-induced protein,” “auxin response factor 18,” “auxin-induced protein 5NG4,” “auxin transporter,” “auxin-binding protein ABP20” and “auxin response factor 8-like.” The expression of these genes can be increased by the development of thicker lateral roots based on environmental demand.

With alterations in rainfall levels in the subtropical seasons, the importance of the ent-kaurene synthase gene must be emphasized as it is linked to viviparity. The ent-kaurene synthase gene is expressed only in the mangroves in the subtropical region and has been reported to be undergoing positive selection in *Rhizophora apiculata* populations (Xu et al., 2017). Viviparity and an increase in rainfall may enable *R. mangle* to facilitate the dispersal and colonization of new subtropical habitats.

Xu et al. (2017) also reported positive selection acting on the SUMO-activating enzyme 2 (SAE2) gene in another Rhizophora species. The expression of SAE2 was found in samples from the subtropical region. The investment of this Rhizophoreae population in specific AA substitutions shows the importance of proteomic modifications to their responses to local selective pressures.

### Response to Low Oxygen, Flooding, Limited Salt Intake and Water Loss

The gene expression profile of each population of *R. mangle* is adapted to the type of environment in which the plant grows. The perpetually soaked soil in which mangroves grow has little available free oxygen. Anaerobic bacteria release nitrogen gas, soluble iron (iron), inorganic phosphates, sulfides and methane, making the soil much less nutritious than other soils. These effects are less intense in subtropical Brazil, where there is less tidal variation. The enrichment of the GO terms “response to oxidative stress” (GO: 0006979), “cell wall” (GO: 0005618) “apoplast” (GO: 0048046) and “N-terminal protein amino acid methylation” (GO: 0006480) in subtropical roots may indicate that there is an intense response to this type of stress even if the tide level is low. High salt concentrations in apoplasts and symplasts represent an advantage for mangrove plants. The enrichment of GO terms related to apoplasts in samples from the subtropical region means that these plants are working to reduce the challenge to the primary walls and cell membranes. These important adaptations for salinity tolerance influence the absorption, transport and loss of water (Reef and Lovelock, 2015).

The response to oxidative stress may be associated with less availability of oxygen in the soil of subtropical mangrove forests than in the soil of equatorial mangrove forests. In addition, as predictions of environmental change indicate that there will be increased flood frequency in the region, this could represent an adaptive resource for the species in this area. Cluster 32 was associated with DETs unique to roots in subtropical samples and with GO terms related to the response to plant oxidative stress, such as “oxidation-reduction process” (GO: 0055114), “microtube-based movement” (GO: 0007018), “cell cycle” (GO: 0007049), “methylation” (GO: 0032259), and “cellular amino acid metabolism” (GO: 0006520) (Wang et al., 2011; Pratelli and Pilot, 2014; Kim et al., 2015).

Cluster 91 was associated with genes in the root that were DETs unique to the subtropical samples and that were associated with many GO terms, including “cuticle development” (GO: 0042335); the cuticle provides a physical barrier against water loss and irradiation and is involved in the activation of pathogen defenses (Serrano et al., 2014). These genes were also associated with the GO terms “light reactions” (GO: 0042548), “photosynthesis” (GO: 0015979), “cellular response to redox state” (GO: 0051775) and “NADH dehydrogenase complex” (GO: 0010258), which are involved in oxidative damage to the photosynthetic machinery and play signaling roles in the regulation of gene expression and protein function in various physiological processes of plants, including acclimatization (Ifuku et al., 2011; Trotta et al., 2014). The enrichment of genes linked to photosynthesis in the samples from the equatorial region indicates that the mangrove roots perform photosynthesis. Some identified genes were also related to glycerol ether metabolism, salt tolerance and “osmotic adjustments” through the accumulation of metabolites in plants (Shen et al., 1999). The presence of these molecular mechanisms that regulate the response to oxidative stress is important in the face of future rising sea levels and flood events in subtropical Brazil.

The roots were the only tissue in which there were more over-expressed than under-expressed genes in the subtropical samples; these genes can be visualized in the network. Root tissue is the tissue most directly affected by oxidative stress; thus, the positive regulation of genes responding to this type of stress in the subtropical samples shows that either the local pollution level or the salinity of the region demands an energy supply.

In turn, the root samples from equatorial mangroves showed a greater investment in ethylene-mediated signaling pathways. There were upregulated “ethylene-responsive transcription” genes in the roots and a unique expression profile of these genes in the stem and flower samples. The enriched genes linked to this signaling pathway play significant roles in salt tolerance (Krishnamurthy et al., 2017).

In addition, GO terms associated with flower and meristem genes in cluster 248 in the equatorial samples were related to “brassinosteroid-mediated signaling” (GO: 0009742), in which brassinosteroids control plant stress responses and regulate the expression of stress response genes through cross-talk with other hormones (Divi et al., 2010).

Cluster 179 was also among the five most representative clusters of the stems, leaves and meristems, and its nodes were linked to genes that were DETs that were unique to equatorial samples. The most representative GO terms associated with this cluster included “nuclear-transcribed mRNA catabolism” (GO: 0000956) and “vesicle-mediated transport pathway” (GO: 0016192), which were already described by the constitutive expression of certain proteins related to increased tolerance to osmotic stress (Mazel et al., 2004). The GO terms “response to auxin” (GO: 0009733) and “potassium ion homeostasis” (GO: 0055075) were related to osmotic adjustments, balancing potassium homeostasis in cell growth, drought stress responses, and a high salinity response (Chao et al., 2013; Jung and McCouch, 2013; Osakabe et al., 2013). A high K+/Na+ ratio in the cytosol is one strategy that some plant genotypes with a high tolerance to soil salinity show, which is in contrast to the ratio found in salt stress-susceptible genotypes (Almeida et al., 2017).

Cluster 179 was associated with genes in stems, leaves and meristems that were DETs unique to equatorial samples and with GO terms related to “nuclear-transcribed mRNA catabolism,” “response to auxin,” and “potassium ion homeostasis,” which are important for the response to oxidative stress. In equatorial cluster 226, genes related to the “regulation of pH” (GO: 0006885) were also observed; these genes are very important given the future predictions of increased salinity in the soil in northern Brazil.

Cluster 17 was associated with the most representative DETs unique to equatorial samples, many of which were associated with the GO term “response to absence of light” (GO: 0009646), which may be related to the lack of light during periods of flooding (Van Veen et al., 2016) or may be a reflection of a closed canopy forest. The molecular mechanisms of the response to low light intensity and other abiotic stresses at more specific levels (GO: 0009646) reveal that *R. mangle*, even in a tropical region with high levels of solar radiation, has genes aimed at directing its capacity for metabolic readjustments induced by light stress (GO: 0006508); “water transport” (GO: 0006833), “lipid metabolism” (GO: 0006629), and “lignin catabolism” (GO: 0046274), which include functional groups of proteins affected by salt stress (Tran et al., 2007; Jeon et al., 2010; Zwack et al., 2016); and “cytokinin metabolism” (GO: 0009690), which increases tolerance to drought, salt and freezing under reduced levels of cytokinin signaling. The genes associated with the GO term “peroxisome fission” (GO: 0016559) were also very prevalent in this cluster. In addition, peroxisomal proliferation is induced in plants by oxidative stress (reactive oxygen species, ROS), UV radiation, saline stress, and even clofibrate (Lopez-Huertas et al., 2000; Sinclair et al., 2009; Mitsuya et al., 2010; Schrader et al., 2012).

The significant enrichment of peroxisomal genes may be very important for the ability to effectively eliminate excess ROS due to stress responses and to protect plant cells from oxidative stress induced by prolonged flooding. Excess ROS may result in significant cellular damage, such as damage to DNA, RNA, and proteins. The upregulated transcription of “glutathione-dependent oxidoreductase” and “glutathione-Y-family DNA polymerases family” in the samples from the equatorial region may play an important role in the effective elimination and repair of damage in Rhizophoraceae mangroves caused by oxidative stress and superoxides under conditions of high salinity, high temperature and high levels of UV radiation (Guo et al., 2017).

High levels of ROS can also delay seed germination, reducing germination rates (Zhang et al., 2011). Therefore, the enrichment of reproductive genes may be essential to improve the reproduction and success of equatorial plants in conditions of flooding (Guo et al., 2017). Transcript annotations with GO terms related to the transduction of plant hormonal signals and MAPK signaling were found with UETs in all tissues from the subtropical region. These hormonal responses indicate that there is also feedback regulation of the ABA concentration that is important for the survival of mangroves because ABA accumulation is induced by environmental stimuli such as salt, oxidative stress and high temperatures (Hong et al., 2018). The variations between the two populations in terms of genetic diversity, migration rate, population size and the strength of the founding effects (Francisco et al., 2018) reinforces the differences in the adaptive genomic strategies employed by *R. mangle* growing in equatorial and subtropical regions. As with other Rhizophora species, the frequent sea level fluctuations associated with climate change may have negatively affected their population sizes and played a dominant role in the evolutionary history of these mangrove trees (Yan et al., 2016).

## Conclusion – Mangrove Conservation

The gene-specific co-expression network for each site indicates that vulnerability to climate change is not a pre-defined condition but is constructed by exposing each population to natural hazards and challenges. In this broad perspective on the need to adapt to environmental challenges, it is possible to observe that each region possesses tools in the genetic network capable of responding differently to increasing pressure from stressors. The resilience of the species includes a set of regional survival strategies to cope with present and future impacts.

Subtropical and equatorial mangroves in Brazil are important sources of mangrove genetic diversity; thus, their conservation is necessary to ensure the survival of the species and of the entire ecosystem to which it belongs. It is important for *R. mangle* to retain the sets of genes required for different adaptive responses to climate change and its consequences.

Maintaining the local diversity of mangroves is more important than relying on conservation management predictions of a complex and highly unstable adaptive system. That is, actions that preserve the environment and its diversity can maintain the capacity of mangroves to react and stabilize their ecosystems even in the face of anticipated climatic changes.

The information presented in the gene co-expression network in this study may highlight genes that are candidates for the study of abiotic stress in other plant species. The gene co-expression network for a tree species developed by this work represents an advanced and promising genetic technique that can be used in the study of wetlands and other environmental stressors and in the discovery and development of heat tolerant varieties of plants.

## Deposited Data

The RNA-Seq datasets generated by the Illumina-GAII platform are available from the NCBI Sequence Read Archive database (SRA; https://www.ncbi.nlm.nih.gov/sra/SRP150858) under experiment accession number SRP150858. **Supplementary Data Sheet 1** provides information on all assembly contigs, normalized read counts (RSEM) for each sequenced sample and functional annotations.

## Author Contributions

AdS and MB designed and coordinated the research. SB and MC performed the experiments and conducted the fieldwork. CdS performed the qRT-PCR experiments. SB, MC, NM, CdS, and MB analyzed the data. SB and AdS wrote the manuscript. All co-authors read and approved the text.

## Conflict of Interest Statement

The authors declare that the research was conducted in the absence of any commercial or financial relationships that could be construed as a potential conflict of interest.

## Footnotes

1 http://www.uniprot.org/

2 http://www.geneontology.org/

3 https://transdecoder.github.io/

4 http://pgrc.ipk-gatersleben.de/misa/misa.html

5 http://cytoscape.org
