# Supplementary material for: Extremophiles as a Model of a Natural Ecosystem: Transcriptional Coordination of Genes Reveals Distinct Selective Responses of Plants Under Climate Change Scenarios
Source: Front Plant Sci. 2018 Sep 19;9:1376. doi: 10.3389/fpls.2018.01376 (PMC6156123; doi:10.3389/fpls.2018.01376)
Supplement: Supplementary file 1 [file Table_1.docx]

Supplementary Material

Extremophiles as a Model of a Natural Ecosystem: Transcriptional Coordination of Genes Reveals Distinct Selective Responses of Plants Under Climate Change Scenarios

Stephanie K. Bajay, Mariana V. Cruz, Carla C. da Silva, Natália F. Murad, Marcelo M. Brandão, Anete P. de Souza*

***Correspondence:** Anete Pereira de Souza: anete@unicamp.br

**Supplementary Table 1.** Characterization of the sampling sites.

| **Sequenced sample ID** | **Latitude (S)** | **Longitude (W)** | **Temperature (°C)** | **Rainfall (mm)** | **Insolation (hours)** | **Relative humidity (%)** | **Climate classification according to the KÖPPEN (1936) criteria** | **Characterization** |
| --- | --- | --- | --- | --- | --- | --- | --- | --- |
| Rm Subtropical 1 | 27.27806 | 48.51941 | 18.68 | 2.4 | 7.1 | 86.12 | Cfa | Tall tree, with thin branches at the border of a preserved forest dominated by *Laguncularia racemosa*. Sampling was performed during the low tide. |
| Rm Subtropical 2 | 27.65799 | 48.52684 | 18.68 | 2.4 | 7.1 | 86.12 | Cfa | Tall tree, with thin branches at the border of a preserved forest dominated by *Laguncularia racemosa*. Sampling was performed during the low tide. |
| Rm Subtropical 3 | 27.65878 | 48.52747 | 18.68 | 2.4 | 7.1 | 86.12 | Cfa | Tall tree plenty of propagules at the border of a degraded forest dominated by *Laguncularia racemosa*. Sampling was performed during the low tide. |
| Rm Equatorial 1 | 00.64616 | 47.26404 | 26.02 | 1.57 | 7.83 | 85.28 | Am | Tall flowering tree in a forest dominated by *Rhizophora* *mangle*. Sampling was performed during the low tide. |
| Rm Equatorial 2 | 00.65697 | 47.26457 | 26.02 | 1.57 | 7.83 | 85.28 | Am | Tall flowering tree in a forest dominated by *Rhizophora* *mangle*. Sampling was performed during the low tide. |
| Rm Equatorial 3 | 00.64804 | 47.26389 | 26.02 | 1.57 | 7.83 | 85.28 | Am | Tall flowering tree in a forest dominated by *Rhizophora* *mangle*. Sampling was performed during the low tide. |
