# Supplementary material for: Extremophiles as a Model of a Natural Ecosystem: Transcriptional Coordination of Genes Reveals Distinct Selective Responses of Plants Under Climate Change Scenarios
Source: Front Plant Sci. 2018 Sep 19;9:1376. doi: 10.3389/fpls.2018.01376 (PMC6156123; doi:10.3389/fpls.2018.01376)
Supplement: Supplementary file 3 [file Table_3.docx]

Supplementary Material

Extremophiles as a Model of a Natural Ecosystem: Transcriptional Coordination of Genes Reveals Distinct Selective Responses of Plants Under Climate Change Scenarios

Stephanie K. Bajay, Mariana V. Cruz, Carla C. da Silva, Natália F. Murad, Marcelo M. Brandão, Anete P. de Souza*

***Correspondence:** Anete Pereira de Souza: anete@unicamp.br

**Supplementary Table 3.** Number of Equally Expressed Transcripts (EET) between samples from both sampling populations.

| **Tissue** | **EET between Equatorial and Subtropical samples** |
| --- | --- |
| Flower | 8,518 |
| Leaf | 6,481 |
| Meristem | 5,822 |
| Root | 11,868 |
| Stem | 6,557 |
