# Supplementary material for: Extremophiles as a Model of a Natural Ecosystem: Transcriptional Coordination of Genes Reveals Distinct Selective Responses of Plants Under Climate Change Scenarios
Source: Front Plant Sci. 2018 Sep 19;9:1376. doi: 10.3389/fpls.2018.01376 (PMC6156123; doi:10.3389/fpls.2018.01376)
Supplement: Supplementary file 4 [file Table_4.docx]

Supplementary Material

Extremophiles as a Model of a Natural Ecosystem: Transcriptional Coordination of Genes Reveals Distinct Selective Responses of Plants Under Climate Change Scenarios

Stephanie K. Bajay, Mariana V. Cruz, Carla C. da Silva, Natália F. Murad, Marcelo M. Brandão, Anete P. de Souza*

***Correspondence:** Anete Pereira de Souza: anete@unicamp.br

**Supplementary Table 4.** Primer pairs used in qRT-PCR reactions for the validation of RNA-seq data analyses.

| **Tissue** | **Putative homologous gene symbol in the reference species** | **Putative homologous gene name in the reference species** | **Left Primer sequence** | **Right Primer sequence** | **Annealing temperature (°C)** |
| --- | --- | --- | --- | --- | --- |
| Leaf | PRS7 | PRS7_PRUPE - 26S protease regulatory subunit 7 | ATCGCAAGGTTGAGTTTGGG | AGTGGAATTTGGGCAAAGGC | 60 |
| Leaf | PGMC | PGMC_POPTN - Phosphoglucomutase, cytoplasmic | TTTTCTCTGTGCAGCCGTAC | GAAGCCACGCAAATAACAAGC | 60 |
| Leaf | DSP8 | Putative dual-specificity protein phosphatase DSP8 | AACGGCTCCTGGATGAATCTC | GAGAAGAAAGCTCGCATTGTGG | 60 |
| Leaf | RGAP7 | RGAP7_ARATH - Rho GTPase-activating protein 7 | AGGTCAGCTATTGCAACGTG | TGCTGTCGCGTCAAGATTAG | 60 |
| Leaf | 4CLL9 | 4CLL9_ARATH - 4-coumarate--CoA ligase-like 9 | TGCTTCAGATGGATGGCTAAGG | AACAAGGCCTCTAGTTCTGCTG | 60 |
| Root | Sphinganine | Sphinganine C4-monooxygenase 1 | GCACATCCATTCTCTGCATCAC | GGCTCCACCAATTGTATCAAGC | 60 |
| Root | At3g53170 | Pentatricopeptide repeat-containing protein At3g53170 | TGAAGGAAGGAATGCAGCTG | AACCAGGTGGATTTCATGCC | 60 |
| Root | At3g61590 | F-box/kelch-repeat protein At3g61590 | GATGTTTTTGCTGGCAGTGG | TGGGGCACTTTTTGTCACTG | 60 |
| Root | ubiquinone | NADH dehydrogenase [ubiquinone] iron-sulfur protein 2 | AACAAGAAGGCGAGCTGATC | AAGCCTCAAAAAGCGAAGGC | 60 |
| Root | OGG1 | N-glycosylase/DNA lyase OGG1 | CCCACAGCTTGTTTCCCTTATC | TGTGGCAAGTAAGGAGTCTCAG | 60 |
