# Supplementary material for: Extremophiles as a Model of a Natural Ecosystem: Transcriptional Coordination of Genes Reveals Distinct Selective Responses of Plants Under Climate Change Scenarios
Source: Front Plant Sci. 2018 Sep 19;9:1376. doi: 10.3389/fpls.2018.01376 (PMC6156123; doi:10.3389/fpls.2018.01376)
Supplement: Supplementary file 5 [file Table_5.docx]

Supplementary Material

Extremophiles as a Model of a Natural Ecosystem: Transcriptional Coordination of Genes Reveals Distinct Selective Responses of Plants Under Climate Change Scenarios

Stephanie K. Bajay, Mariana V. Cruz, Carla C. da Silva, Natália F. Murad, Marcelo M. Brandão, Anete P. de Souza*

***Correspondence:** Anete Pereira de Souza: anete@unicamp.br

**Supplementary Table 5.** Loci selected from the assembled transcriptome for qRT-PCR validation of differential expression analysis results.

| **Putative homologous gene symbol in the reference species** | **Putative homologous gene name in the reference species** | **Reference species** | **Left Primer sequence** | **Right Primer sequence** | **Annealing Temperature (°C)** | **Tissue** | **Product size (bp)** |
| --- | --- | --- | --- | --- | --- | --- | --- |
| DBR | 2-alkenal reductase (NADP(+)-dependent) | *Nicotiana tabacum* | AAACAGATCCACGGTCCTGATC | ACAGGGACAACTTCTGACCAC | 60.0 | Leaf | 133 |
| CesA | Cellulose synthase A catalytic subunit 6 [UDP-forming] | *Arabidopsis thaliana* | TGCCCACAGTGCAAAACAAG | ATAGCCTCCGCAATTTGCTC | 60.0 | Leaf | 149 |
| Fbox Protein | F-box protein At1g78100 (conserved protein) | *Arabidopsis thaliana* | AGATAATGCCTCGCTGATGC | TTGCGTGATTCTCGGGTTTG | 60.0 | Leaf | 135 |
| TBL11 | Protein trichome birefringence-like 11 | *Arabidopsis thaliana* | GCCTCGCTAACCTTTTCCAATC | GCAGTCAGACATTCTCGCAAC | 60.0 | Leaf | 90 |
| EXPA11 | Expansin A1, ALPHA 1.2,EXPA1; Expansin-A1 | *Theobroma cacao; Arabidopsis thaliana* | TCGCTGCAGTTTTCATGGTG | TTCCATACCCACAAGCTCCAC | 60.0 | Leaf | 147 |
| OFP7 | Ovate family protein; Transcription repressor OFP7 | *Vitis vinifera*; *Arabidopsis thaliana* | TTCACTCGTTCTGTGGAGAGC | TATTCCAGTTGCCCGAAACC | 60.0 C | Leaf | 127 |
| IDD5 | Protein indeterminate-domain 5, chloroplastic | *Arabidopsis thaliana* | ATTCCCTGCCAGTTATTGCC | AACAGCGTGGCATGGATATG | 60.0 | Root | 137 |
| ALFIN7 | PHD finger protein ALFIN-LIKE 7 | *Arabidopsis thaliana* | GGAGACCAACTCTGTTGTTGTAC | CCAAAACATTTTGTGGCCCATG | 60.0 | Root | 96 |
| GID1B | Gibberellin receptor GID1B | *Arabidopsis thaliana* | TAGTTGTTGTGGCCGGTTTG | AGTGATCGTTGTTCGGCAAG | 60.0 | Root | 144 |
| SK2 | SKP2A_ARATH - F-box protein SKP2A | *Arabidopsis thaliana* | TGCAGCTTTCATGCATCCAC | ATGCATTAGCCCATGGTTGC | 60.0 | Root | 143 |
| ERD4 | CSC1-like protein ERD4 | *Brassica juncea* | TTCAGACCTCAAGCTCATCCTC | AGCTCTTGAAGTTGCTTGCC | 60.0 | Root | 119 |
