# Supplementary material for: Extremophiles as a Model of a Natural Ecosystem: Transcriptional Coordination of Genes Reveals Distinct Selective Responses of Plants Under Climate Change Scenarios
Source: Front Plant Sci. 2018 Sep 19;9:1376. doi: 10.3389/fpls.2018.01376 (PMC6156123; doi:10.3389/fpls.2018.01376)
Supplement: Supplementary file 8 [file Image_2.pdf]

Supplementary Material

Extremophiles as a Model of a Natural Ecosystem: Transcriptional Coordination of Genes Reveals Distinct Selective Responses of Plants Under Climate Change Scenarios

Stephanie K. Bajay, Mariana V. Cruz, Carla C. da Silva, Natália F. Murad, Marcelo M. Brandão, Anete P. de Souza*

*Corresponding author: Anete Pereira de Souza; anete@unicamp.br

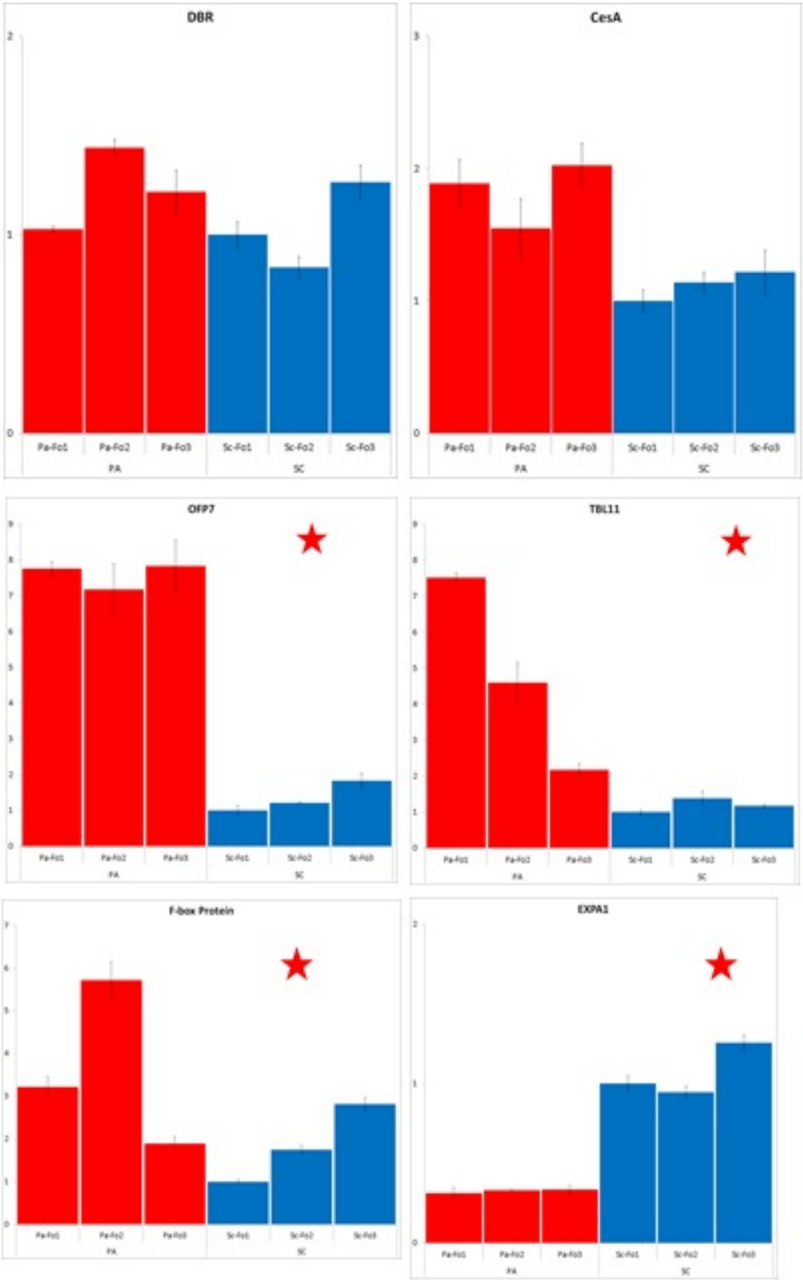

Supplementary Figure 2. Gene expression results for loci amplified in cDNA obtained from leaf samples. The bars represent the mean relative expression of three technical replicates of each individual sample; red represents the tropical samples, and blue represents the subtropical samples. The error bars represent the standard deviation of the mean of the technical replicates. Red stars represent the rejection of the null

hypothesis (the absence of a difference in expression levels between the equatorial and subtropical samples), based on the unpaired Student's t-test with unequal variances, at a significance level of 0.05.
