# Supplementary material for: Extremophiles as a Model of a Natural Ecosystem: Transcriptional Coordination of Genes Reveals Distinct Selective Responses of Plants Under Climate Change Scenarios
Source: Front Plant Sci. 2018 Sep 19;9:1376. doi: 10.3389/fpls.2018.01376 (PMC6156123; doi:10.3389/fpls.2018.01376)
Supplement: Supplementary file 9 [file Image_3.pdf]

frontiers

Supplementary Material

# Extremophiles as a Model of a Natural Ecosystem: Transcriptional Coordination of Genes Reveals Distinct Selective Responses of Plants Under Climate Change Scenarios

Stephanie K. Bajay, Mariana V. Cruz, Carla C. da Silva, Natália F. Murad, Marcelo M. Brandão, Anete P. de Souza*

*Corresponding author: Anete Pereira de Souza; anete@unicamp.br

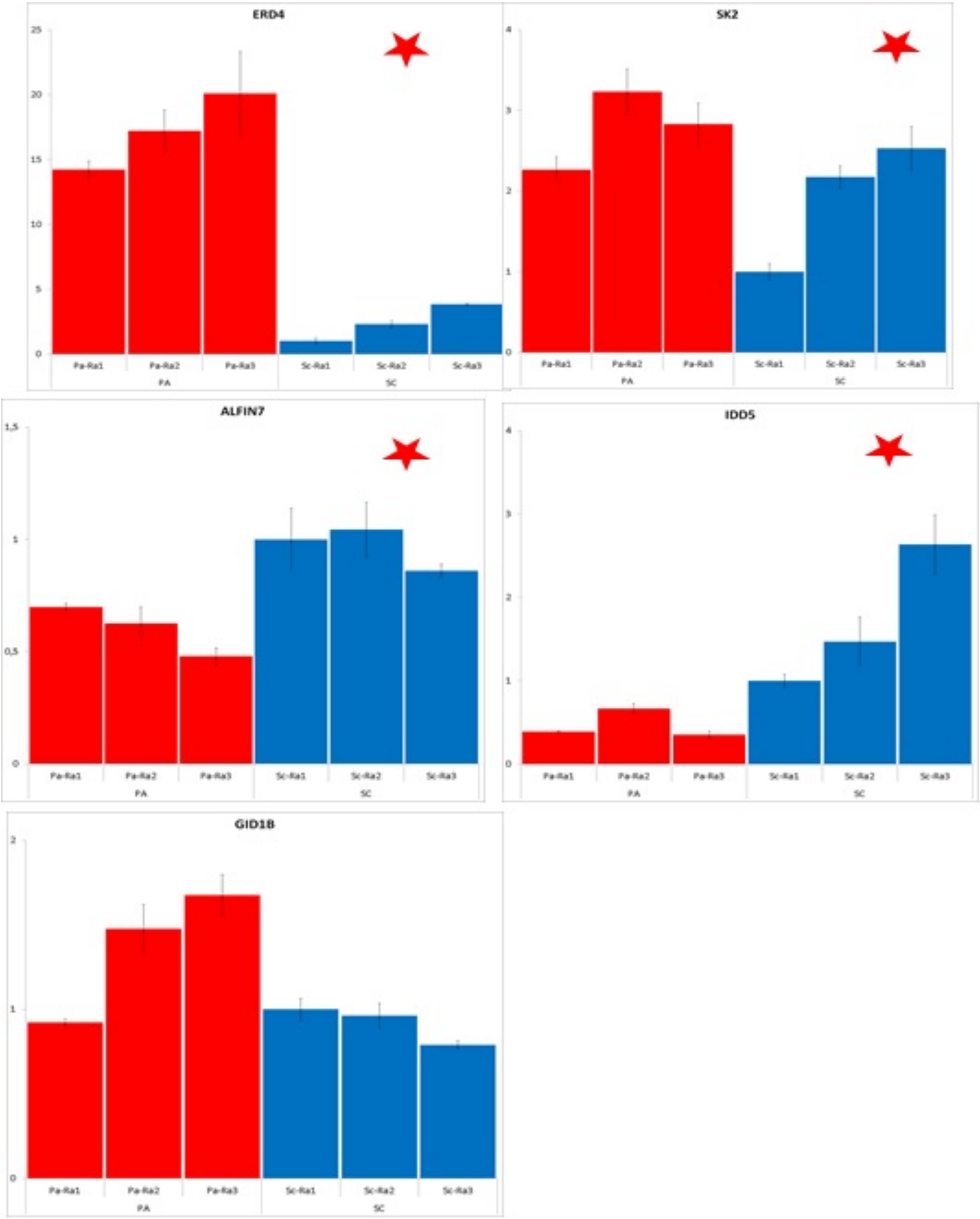


**Supplementary Figure 3.** Gene expression results for loci amplified from cDNA obtained from root samples. The bars represent the mean relative expression of three technical replicates of each individual sample; red represents the tropical samples, and blue represents the subtropical samples. The error bars represent the standard deviation of

the mean of the technical replicates. Student’s t-test was employed to compare the expression levels between the two sampling groups, at a significance level of 0.05, with unequal variance (red stars represent the rejection of the null hypothesis).
