# Supplementary material for: Extremophiles as a Model of a Natural Ecosystem: Transcriptional Coordination of Genes Reveals Distinct Selective Responses of Plants Under Climate Change Scenarios
Source: Front Plant Sci. 2018 Sep 19;9:1376. doi: 10.3389/fpls.2018.01376 (PMC6156123; doi:10.3389/fpls.2018.01376)
Supplement: Supplementary file 11 [file Image_5.pdf]

frontiers

Supplementary Material

Extremophiles as a Model of a Natural Ecosystem: Transcriptional Coordination of Genes Reveals Distinct Selective Responses of Plants Under Climate Change Scenarios

Stephanie K. Bajay, Mariana V. Cruz, Carla C. da Silva, Natália F. Murad, Marcelo M. Brandão, Anete P. de Souza*

***Correspondence:** Anete Pereira de Souza: anete@unicamp.br

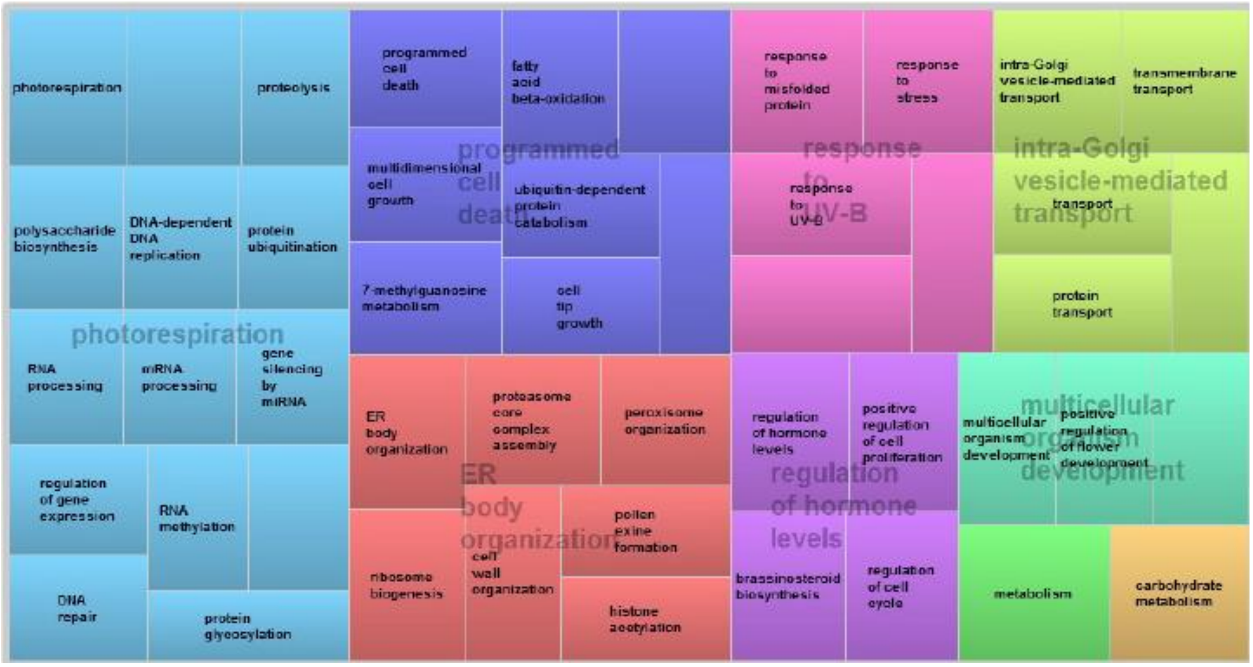

Supplementary Figure 5. Treemap by REVIGO that summarizes the Gene Ontology category biological process, represented excessively by cluster 392 (the fifth most representative of the leaf genes). For the GO term enrichment visualization, a graphical representation (referred to as a treemap) was generated for every network using the GO id and the associated p-values for the enrichment analysis (lower than 0.05), allowing a similarity of 0.9 through REVIGO (Mutwil *et al.*, 2009). The treemap summarizes the ontology terms related to each other based on the hierarchical distance between the terms, their frequency and the absolute log10 p-value.
